# Anticancer activity of MPT0G157, a derivative of indolylbenzenesulfonamide, inhibits tumor growth and angiogenesis

**DOI:** 10.18632/oncotarget.4068

**Published:** 2015-05-27

**Authors:** Yen-Chia Huang, Fang-I Huang, Samir Mehndiratta, Ssu-Chia Lai, Jing-Ping Liou, Chia-Ron Yang

**Affiliations:** ^1^ School of Pharmacy, College of Medicine, National Taiwan University, Taipei, Taiwan; ^2^ School of Pharmacy, College of Pharmacy, Taipei Medical University, Taipei, Taiwan

**Keywords:** HDAC, tumor microenvironment, angiogenesis, HIF-1α, Hsp90

## Abstract

Histone deacetylases (HDACs) display multifaceted functions by coordinating the interaction of signal pathways with chromatin structure remodeling and the activation of non-histone proteins; these epigenetic regulations play an important role during malignancy progression. HDAC inhibition shows promise as a new strategy for cancer therapy; three HDAC inhibitors have been approved. We previously reported that N-hydroxy-3-{4-[2-(2-methyl-1H-indol-3-yl)-ethylsulfamoyl]-phenyl}-acrylamide (MPT0G157), a novel indole-3-ethylsulfamoylphenylacrylamide compound, demonstrated potent HDAC inhibition and anti-inflammatory effects. In this study, we evaluated its anti-cancer activity *in vitro* and *in vivo*. MPT0G157 treatment significantly inhibited different tumor growth at submicromolar concentration and was particularly potent in human colorectal cancer (HCT116) cells. Apoptosis and inhibited HDACs activity induced by MPT0G157 was more potent than that by the marketed drugs PXD101 (Belinostat) and SAHA (Vorinostat). In an *in vivo* model, MPT0G157 markedly inhibited HCT116 xenograft tumor volume and reduced matrigel-induced angiogenesis. The anti-angiogenetic effect of MPT0G157 was found to increase the hyperacetylation of heat shock protein 90 (Hsp90) and promote hypoxia-inducible factor-1α (HIF-1α) degradation followed by down-regulation of vascular endothelial growth factor (VEGF) expression. Our results demonstrate that MPT0G157 has potential as a new drug candidate for cancer therapy.

## INTRODUCTION

It has been known that genetic alterations as well as epigenetic modulations can significantly promote tumor cell progression [1]. Colorectal cancer, the third most common malignant neoplasm worldwide, has shown a sequential accumulation of genetic and epigenetic changes in tumor suppressor genes and oncogenes [2, 3]. Histone deacetylases (HDACs) are the key enzymes of epigenetic regulation by post-translational modifications of core histone or non-histone proteins. Therefore, HDACs are the new and promising anticancer targets.

Communication between cells in tumor progression and the tumor microenvironment is widely thought to be crucial for tumor growth. In particular, the interactions of tumor cells and hypoxia induced angiogenesis play a pivotal role in tumor development [4]. Inflammatory mediators, such as cyclooxygenase-2 (COX-2)-derived prostaglandin E2 (PGE_2_), also have been reported as an important factor for cancer progression and contribute to the redirection of the differentiation of dendritic cells into myeloid-derived suppressor cells [5], recruitment of monocytes/macrophages to tumor sites, and inducing them to differentiate into M2-type tumor-associated macrophages to support tumor angiogenesis and invasion [6, 7]. Recent proteomic analyses have identified an accumulating list of substrates for HDACs; these include a substantial number of key signal transduction components and transcription factors that are involved in tumor microenvironment progression, such as angiogenesis [8], cell cycle progression, and cellular differentiation [9]. This suggests that HDACs exert multiple roles in the tumor microenvironment development. Although there are several novel synthesized compounds that exhibit potent HDAC inhibition and anticancer activity [8–10], the understanding of these anticancer mechanisms in possibly regulating the tumor microenvironment is still limited.

Analyses of these small molecule HDAC inhibitors indicated the *N*-hydroxyacrylamide group plays an important role in the inhibition of HDAC activity [11]. We previously developed a series of indole-3-ethylsulfamoylphenylacrylamides compounds that were based on the core structure of PXD101 (Belinostat) and show apparent anti-inflammatory activity [12]. Among these compounds, we found that N-hydroxy-3-{4-[2-(2-methyl-1H-indol-3-yl)-ethylsulfamoyl]-phenyl}-acrylamide (MPT0G157) exhibited both inhibitory characteristics of HDAC and lipopolysaccharide (LPS)-induced NF-κB signals/cytokines release superior to PXD101 [12]. However, the molecular action of MPG0G157 in the inhibition of cancer growth has not been clearly elucidated. In this study, we examined the anti-tumor activities of MPT0G157 in human colorectal cancer (HCT116) cells, and evaluated the inhibitory effect on tumor growth and angiogenesis.

## RESULTS

### The synthesis of MPT0G157

**Figure 1:**
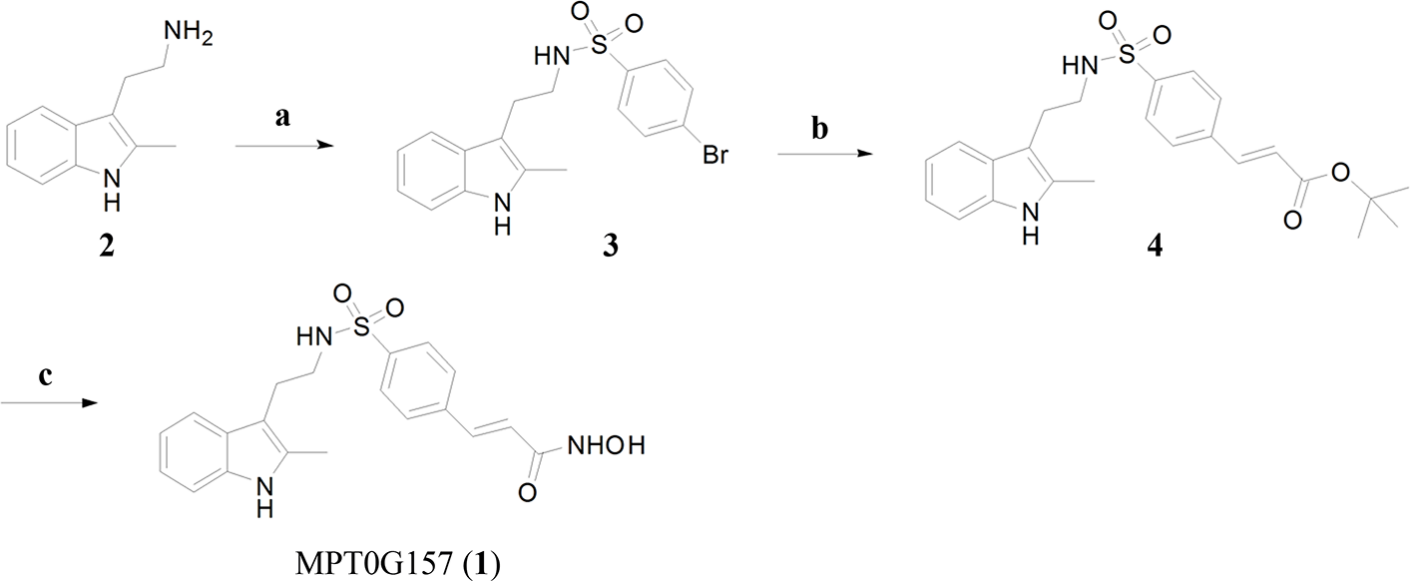
The synthesis of MPT0G157 Reagents and conditions: **A.** 4-bromobenzenesulfonyl chlorides, pyridine, ACN, r.t.; **B.** *t*-Butyl acrylate, Pd_2_(dba)_3_, [(*t*-Bu)_3_P]BF_4_, K_2_CO_3_, TEA, DMF, 100–105°C; **C.** (i) TFA, 0°C to r.t.; (ii) EDC·HCl, HOBt, *N*-methylmorphine, NH_2_OTHP, DMF, r.t.; (iii) 10% TFA, methanol, r.t. Abbreviation: CAN, acetonitrile; DMF, dimethylformamide; K_2_CO_3_, potassium carbonate; Pd_2_(dba)_3_, tris(dibenzylideneacetone) dipalladium; [(*t*-Bu)_3_P]BF_4_, tris-*t*-tubylphosphonium tetrafluoroborate; TFA, trifluoroacetic acid; EDC, 1-Ethyl-3-(3-dimethylaminopropyl)carbodiimide; HOBt, 1-hydroxybenzotriazole; NH_2_OTHP, O-(tetrahydro-2H-pyran-2-yl)hydroxylamine.

The synthesis of MPT0G157 (1) was shown in Figure 1. Briefly, 2-methyltryptamine (2) was allowed to react with 4-bromobenzenesulfonyl chloride in the presence of pyridine and acetonitrile to yield an intermediate compound (3) which was subjected to Heck coupling with *t*-butyl acrylate in the presence of tris(dibenzylideneacetone)dipalladium and tri-*t*-butylphosphonium tetrafluoroborate to yield a cinnamate compound (4). Hydrolysis of the *t*-butyl group in (4) was achieved by treatment with trifluoroacetic acid (TFA). This yielded the corresponding acids which were subject to amidation with O-(tetrahydro-2H-pyran-2-yl)hydroxylamine (NH_2_OTHP) in the presence of 1-Ethyl-3-(3-dimethylaminopropyl)carbodiimide (EDC)·HCl and 1-hydroxybenzotriazole (HOBt), yielding the corresponding protected N-hydroxyamide. Deprotection of the OTHP group was achieved with TFA to yield the compound MPT0G157 (1) in 54% yield. Since the main structure of MPT0G157 was designed and synthesized based on the core structure of PXD101, we used PXD101 as the main reference compound in this study.

### The effects of MPT0G157 on tumor growth suppression and inhibition of HDACs activity

**Figure 2:**
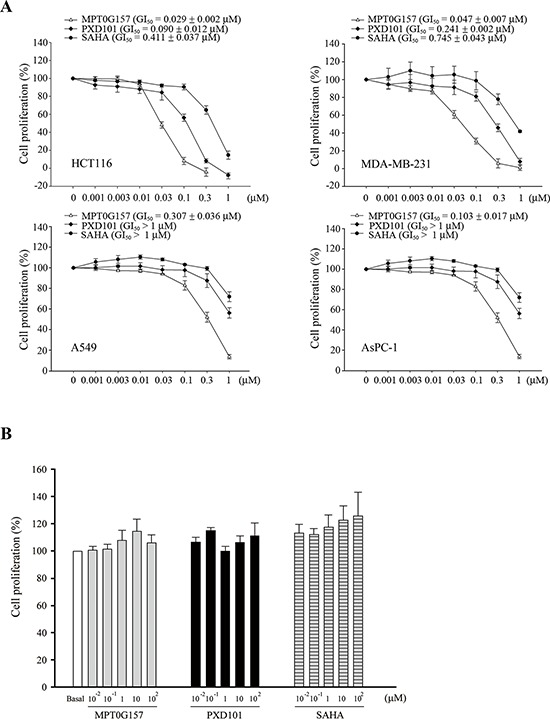
MPT0G157 inhibited tumor growth but less sensitive against normal bone marrow cells **A.** human colorectal cancer (HCT116), breast cancer cell line (MDA-MB-231), lung cancer cell line (A549), pancreatic cancer cell line (AsPC-1) or **B.** human normal bone marrow stromal cells (HS-5) (1 × 10^4^) were incubated with or without indicated concentrations of MPT0G157, PXD101, and SAHA for 48 h. Cell proliferation was evaluated by sulforhodamine B (SRB) assay. Results are shown as mean ± SEM from three independent experiments.

Using HeLa nuclear extract as the HDAC source, our previous study demonstrated that the half maximal inhibitory concentration (IC_50_) of MPT0G157 for inhibition of HDAC activity was 2.8 ± 0.2 nM, which was a more potent inhibitor than PXD101 (IC_50_ = 26.4 ± 1.3 nM) [12]. MPT0G157 also showed anti-inflammatory activity by significantly reducing the production of the inflammatory factors NO, tumor necrosis factor (TNF)-α, interleukin (IL)-6, and PGE_2_, all of which were more potent than PXD101 [12]. We next used the sulforhodamine B (SRB) assay to examine whether MPT0G157 could inhibit cancer growth. MPT0G157 was added to four different kinds of cancer cells: colorectal cancer cell line (HCT116), lung cancer cell line (A549), breast cancer cell line (MDA-MB-231), and pancreatic cancer cell line (AsPC-1), and the GI_50_ value examined. As shown, MPT0G157 treatment inhibited tumor proliferation in the four cancer cell lines in a dose-dependent manner and had the most potent effect in HCT116 cells (GI_50_ = 0.029 ± 0.002 μM). PXD101 and SAHA (Vorinostat) showed less cancer cell growth inhibition (GI) in the four types of cancer cell lines than MPT0G157 (Figure 2A). Therefore, HCT116 was used as the cell line in this study to examine the anti-cancer effect of MPT0G157. We also determined the safety margin of MPT0G157 in human normal cells (bone marrow stromal cells [HS-5]) and found that MPT0G157 was at least 300–3, 400 fold less sensitive against normal cells (GI_50_ > 100 μM), indicating MPT0G157 specifically targets malignant tumor cells (Figure 2B).

**Figure 3:**
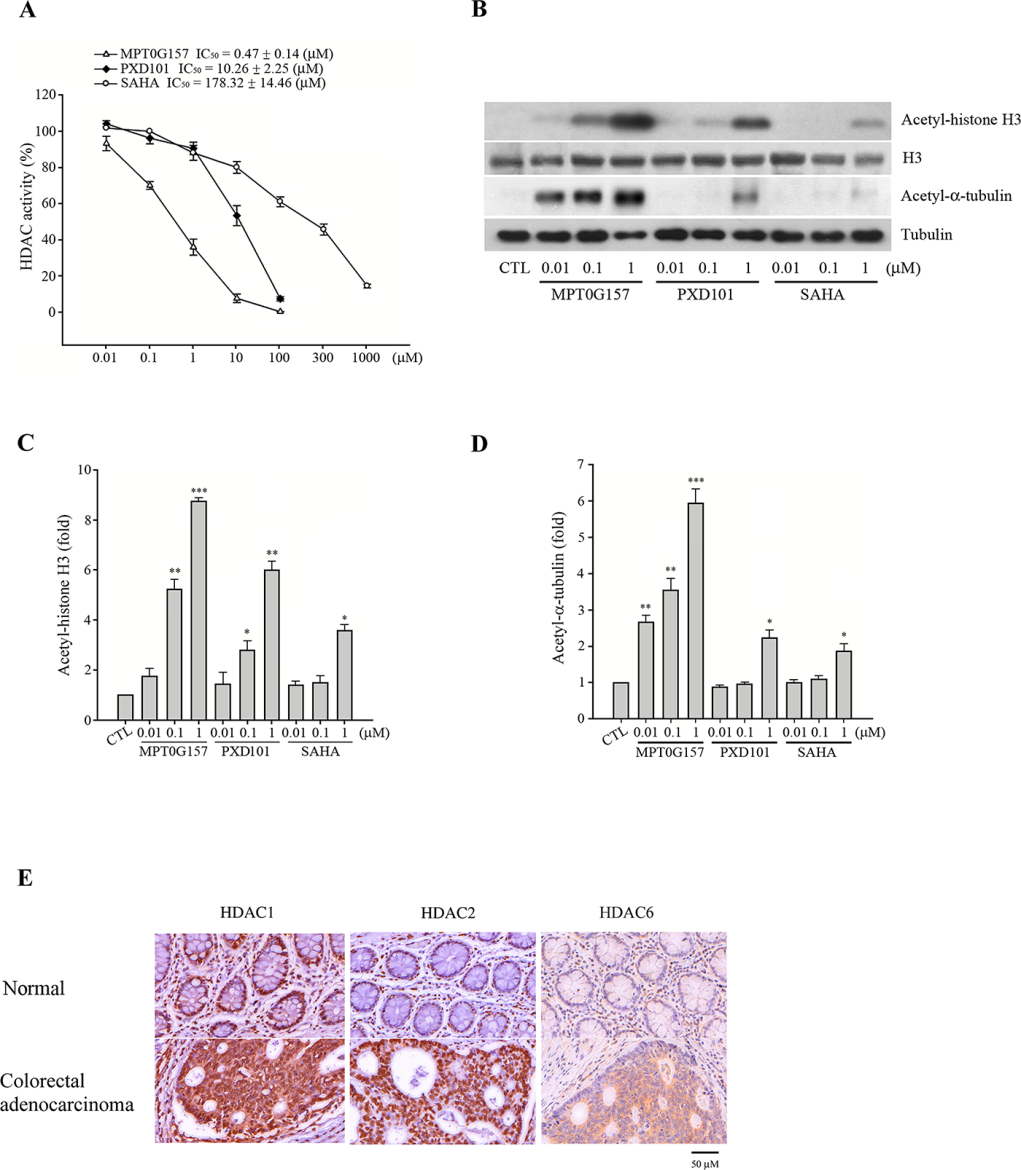
MPT0G157 exhibited potent HDACs inhibitory effect **A.** HCT116 cells (1 × 10^6^) were incubated with indicated concentrations of MPT0G157, PXD101 or SAHA for 24 h, the nuclear proteins were isolated to determine the inhibition of total HDAC enzyme activity. **B.** HCT116 cells (1 × 10^6^) treated as explained in **(A)**, whole-cell extracts were subjected to western blotting for the indicated proteins. Quantitative analysis of acetyl-histone H3 **C.** and acetyl-α-tubulin **D.** expression in western blot were determined by ImageQuant (Molecular Dynamics, USA). **E.** Immunohistochemical analysis of the HDAC1, 2 and 6 expression of a tissue microarray containing normal colon tissues and tumor tissue derived from patients with colon adenocarcinoma. Scale bars are indicated. Results in (A–D) represent mean ± SEM from three independent experiments. *p < 0.05, **p < 0.01, and ***p < 0.001 compared with the control group.

Next, we examined the effect of MPT0G157 on the activity of HDACs by using lysine residues on the substrate, and compared its IC_50_ with PXD101 and SAHA. As shown in Table 1, MPT0G157, at nanomolar concentrations, significantly inhibited HDAC1, 2, and 3 from class I as well as HDAC6 from class IIb. These concentrations were more potent than PXD101 and SAHA. In addition, we noted that MPT0G157 had less activity against recombinant HDAC4 and 8, which was similar to the inhibitory pattern of SAHA. Further, we determined the HDAC inhibitory IC_50_ of MPT0G157 in HCT116 cells. In Figure 3A, the HDAC IC_50_ of MPT0G157 was 0.47 ± 0.14 μM in HCT116 cells, which was more potent than PXD101 (IC_50_ = 10.26 ± 2.25 μM) and SAHA (IC_50_ = 178.32 ± 14.46 μM), respectively. Because histone H3 and α-tubulin are known downstream targets of HDACs, we examined the effects of MPT0G157 on histone H3 and α-tubulin acetylation in HCT116 cells by western blotting. As shown in Figure 3B–3D, MPT0G157 induced a more significant hyper-acetylation of histone H3 and α-tubulin than did PXD101 and SAHA; this result is consistent with its potent inhibitory effect on class I and IIb HDACs. Additional evidence also supported that HDAC1, HDAC2 and HDAC6 over-expression were observed in the stage III colorectal cancer tissues when compared with normal tissues (Figure 3E).

**Table 1:** The inhibition effect of MPT0G157 to individual HDAC isoforms

Compound	IC_50_ (nM ± SEM)					
	Class I				Class IIa	Class IIb
	HDAC1	HDAC2	HDAC3	HDAC8	HDAC4	HDAC6
MPT0G157	7.69 ± 1.84	5.13 ± 0.67	9.36 ± 0.70	>1000	>1000	14.30 ± 0.77
PXD101	43.72 ± 2.56	118.34 ± 9.25	33.21 ± 2.74	218.46 ± 14.58	160.86 ± 2.36	80.31 ± 8.69
SAHA	112.59 ± 12.11	182.87 ± 16.32	232.43 ± 17.52	>1000	>1000	84.75 ± 6.33

### MPT0G157 significantly induced HCT116 cell apoptosis

**Figure 4:**
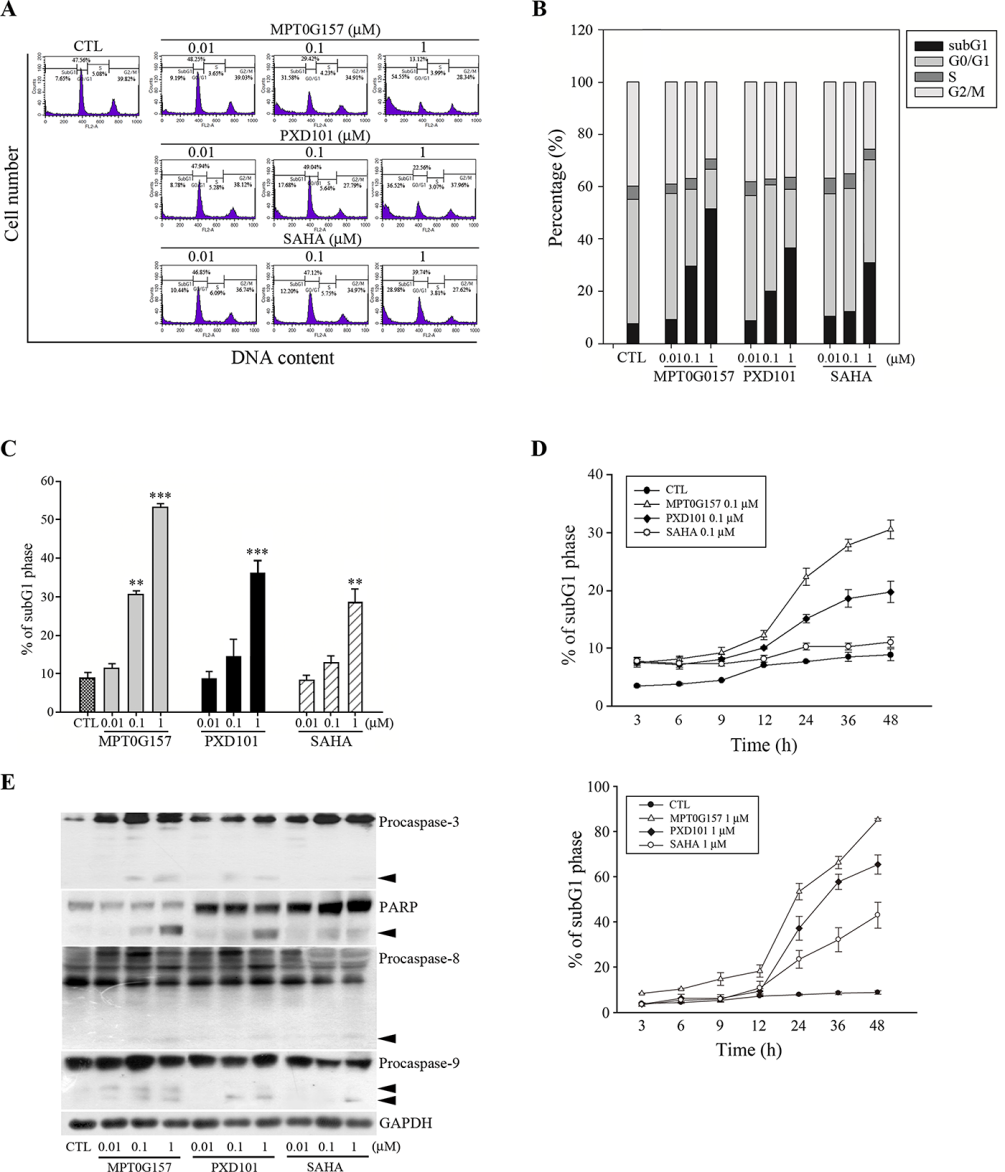
MPT0G157 treatment induced apoptosis in HCT116 cells **A, B.** Cells (1 × 10^6^) were incubated for 48 h with or without MPT0G157, PXD101, and SAHA, fixed and then stained with propidium iodide to analyze (A) the DNA contents by flow cytometry and (B) cell cycle distributions. **C.** Percentages of subG1 phase in response to drug treated as explained in (A) **D.** Time-dependent effects of MPT0G157, PXD101, and SAHA (0.1, 1 μM) on subG1 population. **E.** Cells were incubated as explained in (A), then total cell lysates were prepared for western blot analysis of the indicated proteins; arrowhead indicated the cleavage form of indicated proteins. Results in (C–E) are mean ± SEM from three independent experiments. **p < 0.01, ***p < 0.001 compared with the control group.

Our results have demonstrated that MPT0G157 significantly inhibited tumor growth; next we investigated the effect of MPT0G157 on cell cycle progression. As shown in Figure 4A–4C, treatment with MPT0G157 increased the number and percentage of cells in the sub-G1 phase of the cell cycle in a dose-dependent manner. The reference compounds PXD101 and SAHA also produced a dose-dependent increase in cells in the sub-G1 phase but showed less potency when compared to MPT0G157. MPT0G157 also induced a sub-G1 population in a time-dependent manner while PXD101 and SAHA showed a less potent effect and delayed cytotoxicity (Figure 4D). In addition, MPT0G157 treatment increased caspases-3, -8, and -9, and poly (ADP-ribose) polymerase (PARP) cleavage form expression, suggesting that MPT0G157 induced apoptosis through a caspase-dependent pathway (Figure 4E and Supplementary Figure 3).

### MPT0G157 inhibited growth of human colon cancer cells *in vivo*

**Figure 5:**
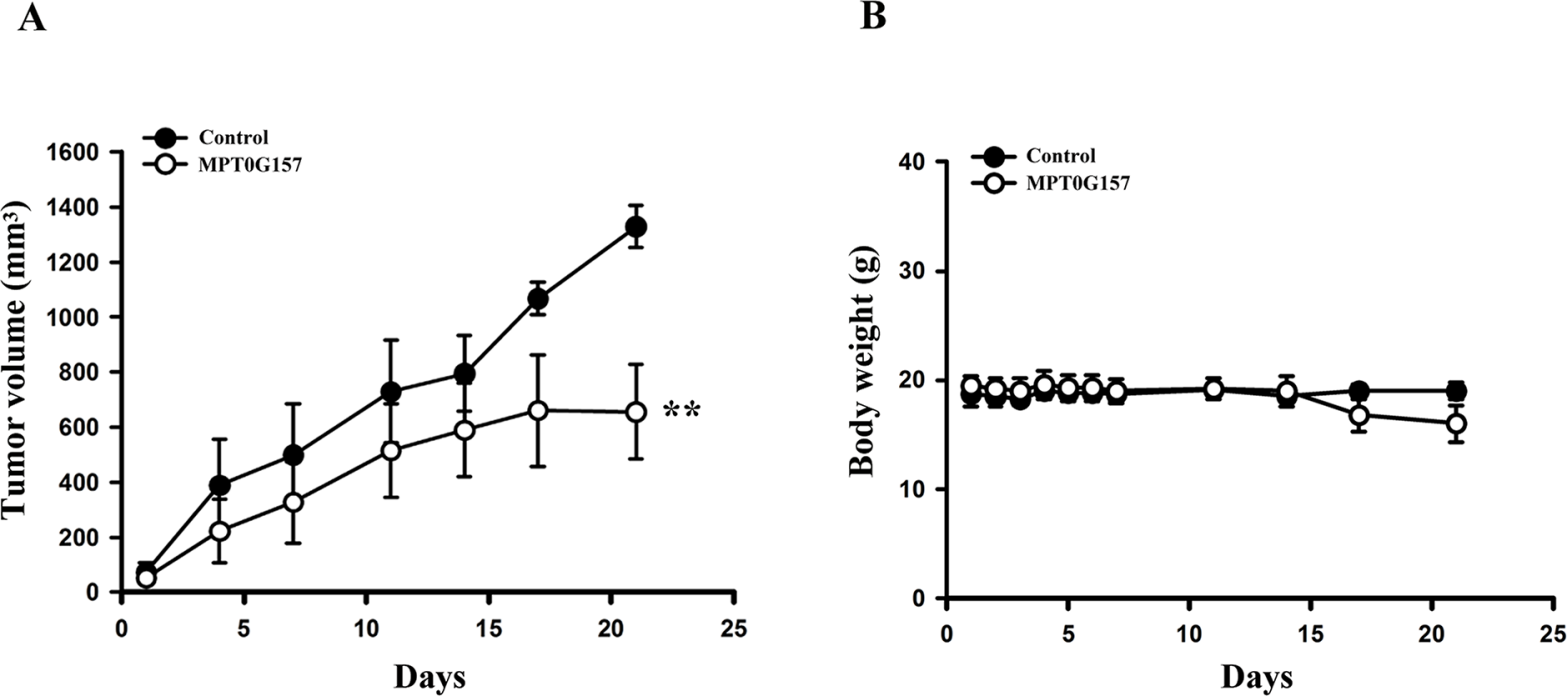
The effect of MPT0G157 in HCT116 xenograft model Mice bearing established HCT116 tumors (~100 mm^3^) were divided into three group (*n* = 5) and dosed as Materials and Methods. The tumor volumes **A.** and body weight **B.** of mice were measured. Results are mean ± SEM. **p < 0.01 versus control group.

Further, we evaluated the *in vivo* inhibitory tumor growth effect of MPT0G157 using a xenograft model. Once a tumor size of 100 mm^3^ was achieved, mice were injected with vehicle (control), or vehicle with MPT0G157 (15 mg/kg) and allowed to reach the endpoint tumor volume of 1, 200 mm^3^. As shown in Figure 5A, administration of MPT0G157 (15 mg/kg) significantly reduced tumor volume. The percent of tumor growth inhibition (TGI) of MPT0G157 was 50.7%. In addition, no significant differences in weight loss were observed during MPT0G157 treatment periods (Figure 5B). In addition, tumor homogenates showed MPT0G157 treatment group markedly reduced COX-2 and phosphorylation of p65 levels while comparing with control group (Supplementary Figure 1). These results demonstrated that MPT0G157 treatment significantly inhibited tumor growth *in vivo*, and this inhibition correlated to its anti-inflammatory effect.

### MPT0G157 treatment suppressed hypoxia-inducible factor-1α response to hypoxia

**Figure 6:**
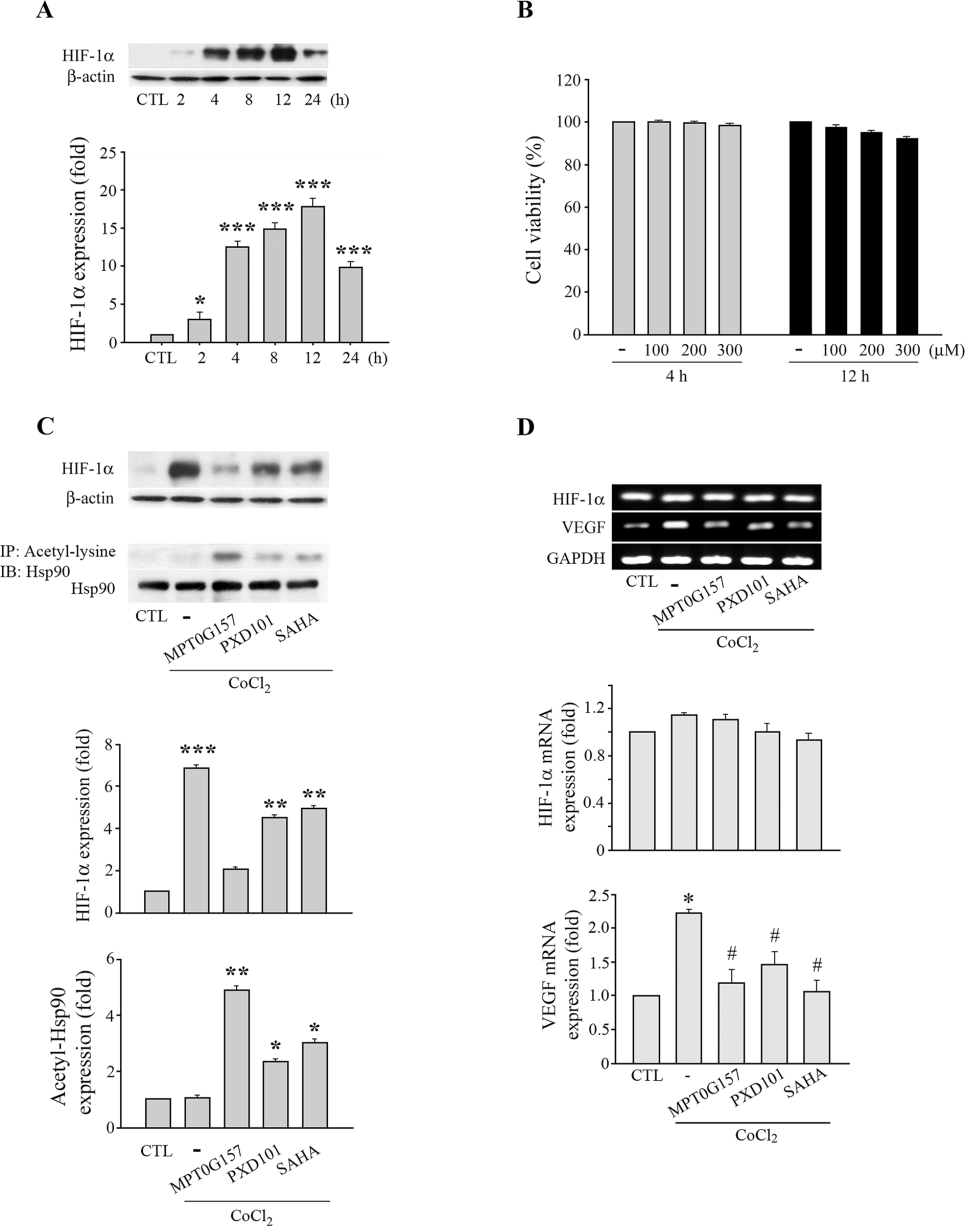
MPT0G157 treatment inhibited HIF-1α expression in HCT116 cells **A.** HCT116 cells (1 × 10^6^) treated with Cobalt(II) chloride (300 μM) for different period as indicated, whole-cell extracts were subjected to western blotting. **B.** Cell viability was determined after 4 or 12 h of treatment with 100–300 μM of Cobalt(II) chloride using the MTT assay (compared with the control group). **C.** Cells were incubated with MPT0G157, PXD101 or SAHA (0.1 μM) for 24 h prior to Cobalt(II) chloride (300 μM) treatment for another 4 h, and total cell lysates were subjected to western blotting for the HIF-1α, Hsp90, β-actin or immunoprecipitated with 1 μg of an anti-acetyl-lysine antibody and immunoblotted for Hsp90 antibody. **D.** Cells were incubated as explained in (C), the levels of hypoxia-inducible factor-1α (HIF-1α), vascular endothelial growth factor (VEGF) mRNA were measured by RT-PCR. Results are shown as mean ± SEM from three independent experiments. *p < 0.05, **p < 0.01, and ***p < 0.001 compared with the relevant control group; #*p* < 0.05 compared with Colbalt(II) chloride-only group.

**Figure 7:**
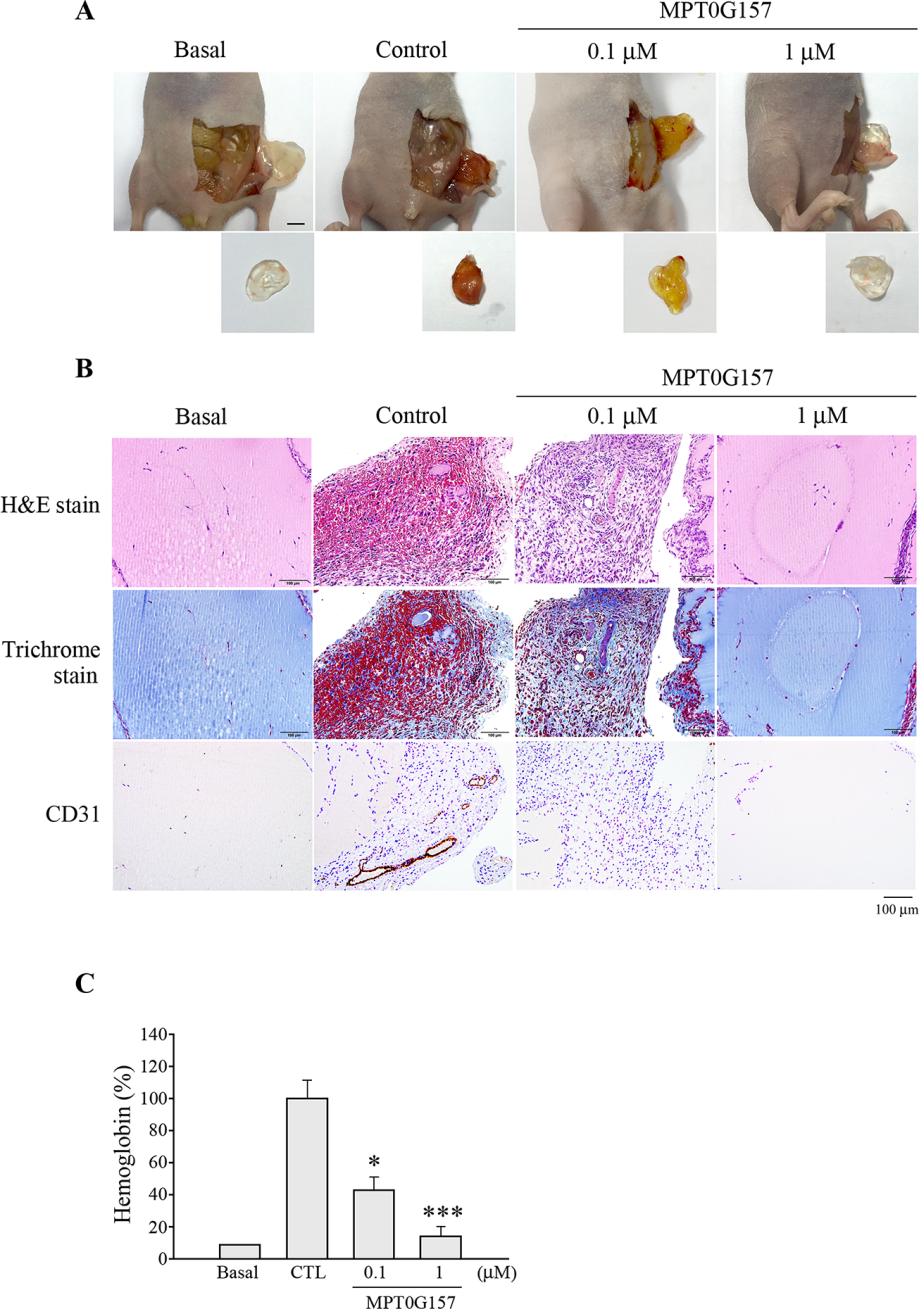
MPT0G157 treatment inhibited the *in vivo* angiogenesis Nude mice were subcutaneously injected with matrigel mixed with or without MPT0G157 (0.1 and 1 μM) (*n* = 3). **A.** After seven days, the animals were sacrificed and the plugs were excised from the mice and photographed. Scale bar represents 0.5 cm. **B.** Sections of H&E, Masson's trichrome stained and CD31 stained matrigel plugs were examined by light microscopy under 200 × magnification. Scale bar = 100 μm. **C.** Quantification of the hemoglobin contents of matrigel plugs by spectrophotometer measured at 540 nm. Data represent the mean ± SEM. *p < 0.05 and ***p < 0.001 versus control group.

We further evaluated the effects of MPG0G157 on angiogenesis, with emphasis on the hypoxia-inducible factor-1α (HIF-1α) response to hypoxia. Cobalt(II) chloride (CoCl_2_), a chemical inducer of hypoxia, can induce HIF-1α expression; we evaluated the inhibition of HIF-1α expression by MPT0G157 in response to CoCl_2_ treatment. As shown in Figure 6A, HCT116 cells that were treated with 300 μM CoCl_2_ for 4 h exhibited significant HIF-1α expression and sustained this expression up to 24 h. The 3-(4, 5-dimethylthiazol-2-yl)-2, 5-diphenyl tetrazolium bromide (MTT) assay demonstrated that CoCl_2_ treatment did not cause cell death (Figure 6B). Furthermore, CoCl_2_ treatment not only significantly induced HIF-1α protein (Figure 6C) but also raised subsequent vascular endothelial growth factor (VEGF) mRNA expression in the HCT116 cells (Figure 6D). MPT0G157 treatment markedly inhibited CoCl_2_-induced HIF-1αprotein (Figure 6C) and VEGF mRNA expressions but did not reduce HIF-1α mRNA levels (Figure 6D), suggesting that MPT0G157 inhibited HIF-1α expression at the post-transcription level. Reference compounds PXD101 and SAHA also reduced HIF-1αprotein and VEGF mRNA levels but were less effective when compared to MPT0G157. Because the newly synthesized HIF-1α molecules need to interact with the chaperone Hsp90 to complete its maturation and stabilization [13, 14], HDAC inhibitor treatment resulted in hyper-acetylation of Hsp90, and further appeared to cause disruption of Hsp90-mediated folding of HIF-1αleading to its subsequent degradation by proteasome [14]. Therefore, we investigated the acetylation of Hsp90 in response to MTP0G157 treatment. As shown in Figure 6C, the amount of Hsp90 acetylation was significantly increased, with a concurrent decrease in HIF-1αand VEGF levels, in response to MPG0G157 treatment. PXD101 and SAHA also increased the Hsp90 acetylation but showed less potent effects. These results demonstrated that MPT0G157 down-regulated HIF-1α and VEGF expressions. We further evaluated the *in vivo* anti-angiogenesis effect of MPT0G157. As shown in Figure 7A, treatment with matrigel containing growth factors significantly increased neovessels than basal group; MPT0G157 down-regulated angiogenesis in a dose-dependent manner. The plugs performed H&E, Masson's trichrome stain and CD31 stain also demonstrated that MPT0G157 inhibited angiogenesis (Figure 7B). Quantitation of angiogenesis by hemoglobin content showed that MPT0G157 significantly suppressed the angiogenic response when compared with the control group (Figure 7C). Results from our studies indicate that MPT0G157, a novel pan-HDAC inhibitor, exhibited potent tumor microenvironment inhibition and also suggest that it has great potential as a new chemotherapeutic agent.

## DISCUSSION

The components of the tumor microenvironment include immune cells, extra-cellular matrices (ECM), and accessory fibroblasts. Reports about the influence of the tumor microenvironment on cancer cells are becoming more prevalent in recent literature. It has been shown that tumors can remodel the stroma and secrete factors that recruit both inflammatory cells and active stromal cells to establish a permissive microenvironment; in turn, cells in the microenvironment produce soluble factors (such as cytokines) that regulate growth and survival of tumor cells [1]. The serum levels of inflammatory factors, including TNF-α, IL-8, and IL-6 are elevated in colorectal carcinoma patients, and it has been suggested that increased plasma levels of these cytokines may have a prognostic role [15]. Supporting studies also indicate that PGE_2_, one of the inflammatory factors, not only plays a significant role in promoting tumor growth but also contributes to a shift in the tumor microenvironment from anti-tumor responses to immunosuppressive responses [6, 16]. Aspirin use resulted in a prolonged overall survival, particularly among colorectal cancer patients whose tumors showed over-expression of COX-2 [17]. Recent studies have identified more various of key signal transduction components and transcription factors that are involved in regulation of tumor microenvironment development, e.g. cell proliferation, survival and angiogenesis, are the substrates of HDACs [8, 9]; suggest that HDACs exerted multiple roles in the tumor progression. Therefore, HDAC inhibitors are considered as promising anticancer agents. Several HDAC inhibitors have been approved by United States Food and Drug Administration (FDA) for the treatment of patients with cutaneous and peripheral T-cell lymphoma, and are undergoing clinical trials for the treatment of solid tumors and leukemia. Our prior study described a series of indole-3-ethylsulfamoylphenylacrylamides derivatives which possessed sulfonamide linkages, the main structural feature of PXD101. These derivatives had been synthesized and evaluated for their effects on HDAC inhibition and inflammatory factors suppression [12]. Among them, MPT0G157 exhibited potent HDAC inhibition (9.4-fold) and suppression of inflammatory factor activities (3.0–25.1-fold) when compared with PXD101 [12]. Therefore, in this study we evaluated whether MPT0G157 could exert anti-cancer activity. We found that MPT0G157 induced growth inhibition in four human cancer cell lines. MPT0G157 induced potent growth inhibition in HCT116 cancer cells but not in normal bone marrow cell growth, and produced a significantly higher percentage of sub-G1 phase and significant caspases-3, -8, and -9, and PARP activation. When all of the results are combined to form a complete picture, it appears that MPT0G157 inhibited HCT116 cancer cell growth and induced apoptosis.

We also observed that MPT0G157 exerted potent inhibition on the activity of HDAC1, 2, and 3 in class I, HDAC6 in class IIb as well as consistently induces acetylation of histone H3 and α-tubulin. Class I and class IIb HDACs have been shown to be over-expressed in human colorectal cancer cells [18, 19], and these two HDACs may contribute to the regulation of cancer cell proliferation, differentiation, and apoptosis by either epigenetic or non-epigenetic modification to modulate p21, p27, cyclins, death receptors or proapototic proteins (e.g. Bim, Bax and Bak) expressions [20–26]. Our results also demonstrated HDAC1, 2 and 6 over-expressions in colorectal adenocarcinoma tissues. Therefore, inhibition of HDACs activity can lead to cancer cell death by the induction of apoptotic pathways [9]. Furthermore, transfected with HDAC1 and HDAC6-encoding plasmids to HCT116 cells not only significantly increased the GI_50_ value, caused the proliferation versus concentration curve right shift, down-regulated subG1 phase percentage, but also reversed the HIF-1α inhibition (Supplementary Figure 2). These evidences demonstrated that the HDACs activity inhibition of MPT0G157 played an important role in its cell proliferation suppression, apoptosis, and HIF-1α reduction. In addition, we also examined the efficiency of MPT0G157 on inhibition of HCT116 cell growth *in vivo*; MPT0G157 significantly inhibited tumor volume. Based on these results, MPT0G157 was shown as a possible novel HDAC inhibitor with potent anti-tumor properties.

The transcription factor HIF-1α plays a key role in cellular adaptations to hypoxic conditions. The degradation of HIF-1α is predominantly mediated by the Von Hippel–Lindau tumor suppressor protein (pVHL) which hydroxylates two prolyl residues (P402 and P564) in the oxygen degradation domain (ODD) of HIF-1α [27]. In normoxia, HIF-1α is rapidly degraded via the ubiquitin-proteasome system by a process that depends on the interaction between the HIF-1α-ODD and pVHL [14, 27]. However, a recent study indicated that HDAC regulated HIF-1α degradation through an ubiquitin-independent proteasomal degradation [14]. Treatment with the HDAC inhibitor, trichostatin A, or silencing of HDAC6, disrupts the Hsp90-mediated folding of HIF-1α and lead to its degradation, suggesting that an ubiquitin-independent pathway was involved in HIF-1α degradation [14]. Supporting evidence also suggests that HDAC inhibitors treatment or suppression of HDAC6 expression induces hyper-acetylation of Hsp90, inhibits its chaperone function, and accelerates client proteins degradation [28]. Consistent with these results, our data showed MPT0G157 treatment significantly down-regulated CoCl_2_-induced HIF-1α and VEGF expression at the post-transcription level since MPT0G157 treatment only inhibited CoCl_2_-induced HIF-1αprotein (Figure 6C) expressions but did not reduce HIF-1α mRNA levels (Figure 6D); and similar observations were reported in previous studies [14, 29]. In addition, MPT0G157 treatment significantly down-regulated Cobalt(II) chloride-induced HIF-1α protein, and this down-regulation can be reversed by MG132 (a proteasome inhibitor) treatment (Supplementary Figure 4). This result demonstrated that MPT0G157 treatment resulted in hyper-acetylation of hsp90 and further caused disruption of HIF-1α, leaded to its subsequent degradation by proteasome. Furthermore, this down-regulation correlated with MPT0G157 mediated HDAC6 inhibition by increasing Hsp90 acetylation, suggesting that MPT0G157 possessed anti-angiogenetic potential; and these effects were unlike to our previous developed compound to increase p21 expression, redistribute E-cadherin and activate PKC to induce cell apoptosis and differentiation [9]. We also determined the anti-angiogenic effect of MPT0G157 *in vivo*. Our results showed that MPT0G157 significantly down-regulated the angiogenic response when compared with the control group. In addition, pharmacokinetics and toxicology profiles will be evaluated in the future for further application.

Taken together, our study indicates that MPG0G157, a novel HDAC inhibitor, not only possessed potent cytokines release suppression effect but also exhibited tumor growth inhibition and anti-angiogenesis. These results are indicative of its possible therapeutic potential in cancer treatment.

## MATERIALS AND METHODS

### Materials

MPT0G157, PXD101, and SAHA were synthesized by Professor Jing-Ping Liou, and the purities were > 98%. We used non-conjugated primary antibodies against HDAC6, Caspases-3, -8 and -9, PARP, acetyl-histone 3, histone 3, acetyl-α-tubulin, α-tubulin, HIF-1α, Hsp90, acetyl-lysine and CD31 (Cell Signaling Technology, Danvers, MA, USA); HDAC1 and HDAC2 antibodies were purchased from Abcam (Cambridge, MA, USA). The labeled secondary antibodies were horseradish peroxidase (HRP)-conjugated anti-mouse or anti-rabbit IgG antibodies (Jackson ImmunoResearch Inc., West Grove, PA, USA). Unless otherwise stated, all other chemicals were purchased from Sigma-Aldrich (St. Louis, MO, USA).

### Cell culture

HCT116, MDA-MB-231, A549, and AsPC-1 were purchased from Bioresource Collection and Research Center (Hsinchu, Taiwan). The human bone marrow stromal cell line HS-5 was kindly provided by Prof. Yu, Alice Lin-Tsing (Genomics Research Center, Academia Sinica, Taipei, Taiwan). The cells were cultured in Roswell Park Memorial Institute medium (RPMI)1640 (HCT116, MDA-MB-231 and AsPC-1) or Dulbecco's Modified Eagle's medium (DMEM) (A549 and HS-5) respectively supplemented with 10% (v/v) heat-inactivated fetal bovine serum (both from Invitrogen^TM^ Life Technologies, Carlsbad, CA, USA), 100 U/mL of penicillin, and 100 μg/mL of streptomycin (Biological Industries, Kibbutz Beit Haemek, Israel). All cells were maintained at 37°C in a humidified atmosphere of 5% CO_2_ in air.

### Cell cytotoxicity and cell proliferation assay

Cell cytotoxicity was measured by the colorimetric MTT assay. Cells (1 × 10^4^) in 100 μL of medium in 96-well plates were incubated with vehicle (control) or vehicle with test compound for 12 or 24 h. After various treatments, 1 mg/mL of MTT was added and the plates were incubated at 37°C for an additional 2 h, then the cells were pelleted and lysed in 100 μL of dimethyl sulfoxide, and the absorbance at 550 nm was measured on a microplate reader. Cell proliferation was measured by the SRB assay. Cells (1 × 10^4^) were incubated for 48 h with the indicated concentrations of test compounds, then were fixed with 10% trichloroacetic acid, stained for 30 min with SRB (0.4% in 1% acetic acid), and washed repeatedly with 1% acetic acid. Protein-bound dye was dissolved in 10 mM Tris base solution and the optical density at 510 nm measured.

### RT-PCR analysis

Total RNA was isolated from cells using TRIzol reagent (Invitrogen). Single-strand cDNA for a polymerase chain reaction (PCR) template was synthesized from 5 μg of total RNA using random primers and Moloney murine leukemia virus reverse transcriptase (Promega). The following oligonucleotide primers were used for amplification: for human HIF-1α (GenBank Accession No. NM_001243084.1), 5′-AGT GTA CCC TAA CTA GCC GAG GAA-3′ (forward) and 5′-CTG AGG TTG GTT ACT GTT GGT ATC-3′ (reverse); for human VEGF-A (GenBank Accession No. NM_001287044.1), 5′-TGC AGA TTA TGC GGA TCA AAC C-3′ (forward) and 5′-TGC ATT CAC ATT TGT TGT GCT GTA G-3′ (reverse); glyceraldehyde-3-phosphate dehydrogenase (GAPDH) (GenBank Accession No. NM_002046), 5′-ATT CCA CCC ATG GCA AAT TC-3′ (forward) and 5′-TGG GAT TTC CAT TGA TGA CAA G-3′ (reverse). Equal amounts (1 μg) of each reverse-transcription product were PCR-amplified using *Taq* polymerase and 35 cycles of 1 min at 95°C, 1 min at 58°C, and 1 min at 72°C. The amplified cDNA was run on 1% agarose gels and visualized under UV light following staining with SYBR Safe DNA gel stain (Invitrogen).

### Immunohistochemical analysis of tissue microarrays

Tissue microarrays containing normal colon tissues and tumor tissue derived from patients with colon adenocarcinoma were purchased from SuperBioChips (Seoul, Korea) and were analyzed for HDAC1, 2 and 6 expressions by immunohistochemical staining.

### Immunoblot and immunoprecipitation analyses

Cells (1 × 10^6^) were incubated for 10 min at 4°C in lysis buffer (20 mM HEPES, pH 7.4, 2 mM EGTA, 50 mM β-glycerophosphate, 0.1% Triton X-100, 10% glycerol, 1 mM DTT, 1 μg/mL of leupeptin, 5 μg/mL of aprotinin, 1 mM phenylmethylsulfonyl fluoride, and 1 mM sodium orthovanadate), were scraped off, incubated on ice for an additional10 min, and centrifuged at 17, 000 g for 30 min at 4°C. Protein samples (80 μg) were then electrophoresed on sodium dodecyl sulfate polyacrylamide gels (SDS-PAGE) and transferred onto a nitrocellulose membrane, which was then blocked by incubation for 30 min at room temperature with 5% fat-free milk in phosphate-buffered saline (PBS). Immunoblotting was performed by overnight incubation at 4°C with primary antibodies in PBS, followed by incubation for 1 h at room temperature with HRP-conjugated secondary antibodies. Bound antibodies were measured using ECL reagent (Advansta Corp., Menlo Park, CA, USA) and exposure to photographic film. In the immunoprecipitation assay, cell lysates (120 μg) were immunoprecipitated overnight at 4°C with 1 μg of anti-acetyl-lysine antibody and A/G agarose beads. The precipitated beads were washed three times with 1 mL of ice-cold cell lysis buffer and bound immune complexes separated by 8% SDS-PAGE, followed by immunoblotting using the anti-Hsp90 antibody.

### Total HDAC enzymatic activity assay

Cells were treated with either vehicle or vehicle with test compounds for 24 h. Total cell lysates were collected and the total HDAC enzyme activity was analyzed using HDAC activity fluorometric assay kit (Cat No. K330–100; BioVision, Milpitax, CA, USA). A fluorescence plate reader with excitation at 355 nm and emission at 460 nm was used to quantify HDAC activity. IC_50_ was determined at the drug concentration that results in 50% reduction of total HDAC activity when compared with the control group.

### Inhibition of recombinant HDAC enzyme activity

Fluorogenic HDAC assay kits (BPS Bioscience Corp., San Diego, CA, USA) were used to assess the ability of HDAC inhibitors to inhibit deacetylation of lysine residues on the substrate by recombinant HDAC1, 2, 3, 4, 6, or 8 according to the manufacturer's instructions.

### Xenograft studies

HCT116 cells were implanted subcutaneously into four week old male nude mice. When the tumors reached the average volume of 100 mm^3^, the mice were randomly divided into three groups (*n* = 5) and then were treated with the vehicle (1% carboxymethyl cellulose + 0.5% Tween 80, 0.2 mL/mouse), MPT0G157 (15 mg/kg) by intravenously injection five on two off in the first week and intraperitoneal injection in the other weeks. The length (L) and width (W) of the tumor were measured by caliper every 3 to 4 days, and the tumor volume was calculated as L × W^2^/2.

### *In vivo* matrigel plug assay

Six-week-old female nude mice were divided into four groups with three animals in each group. Mice were injected subcutaneously into the abdomens with matrigel (BD Bioscience) mixed with PBS or angiogenic factors (40 ng/ml) with/without MPT0G157 (0.1 μM and 1 μM). After seven days, the animals were sacrificed and the plugs were carefully dissected and photographed, the plugs were performed hematoxylin and eosin (H&E), Masson's trichrome stain and CD31 staining. To quantify the blood vessel formation, hemoglobin content was analyzed by Drabkin's reagent kit (Sigma Chemical, St. Louis, MO).

### Ethics

Animal experiments were approved by the Institutional Animal Care and Use Committee of the National Taiwan University College of Medicine (IACUC number: 20110303).

### Data analysis

The data are expressed as the mean ± SEM and were analyzed using one-way ANOVA. When ANOVA showed significant differences between groups, Tukey's post hoc test was used to determine the pairs of groups showing statistically significant differences. A *p* value < 0.05 was considered statistically significant.

## SUPPLEMENTARY FIGURES


