# Supplementary material for: Anticancer activity of MPT0G157, a derivative of indolylbenzenesulfonamide, inhibits tumor growth and angiogenesis
Source: Oncotarget. 2015 May 27;6(21):18590–601. doi: 10.18632/oncotarget.4068 (PMC4621912; doi:10.18632/oncotarget.4068)
Supplement: Supplementary file 1 [file oncotarget-06-18590-s001.pdf]

SUPPLEMENTARY FIGURES

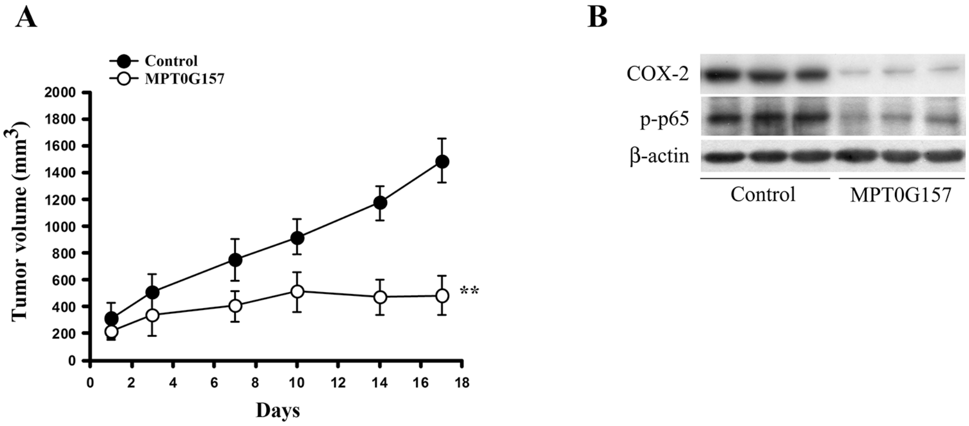

Supplementary Figure 1: MPT0G157 significantly reduced tumor volume and inflammatory factors expression in HCT116 xenograft model. Mice bearing established HCT116 tumors (~100 mm^3) were divided into control and MPT0G157 treatment group ($n = 3$) and dosed as Materials and Methods. The tumor volumes **A.** of mice were measured. **B.** HCT116 xenograft tumor homogenates were analyzed by western blots. Results are mean ± SEM. $^{**}p < 0.01$ versus control group.

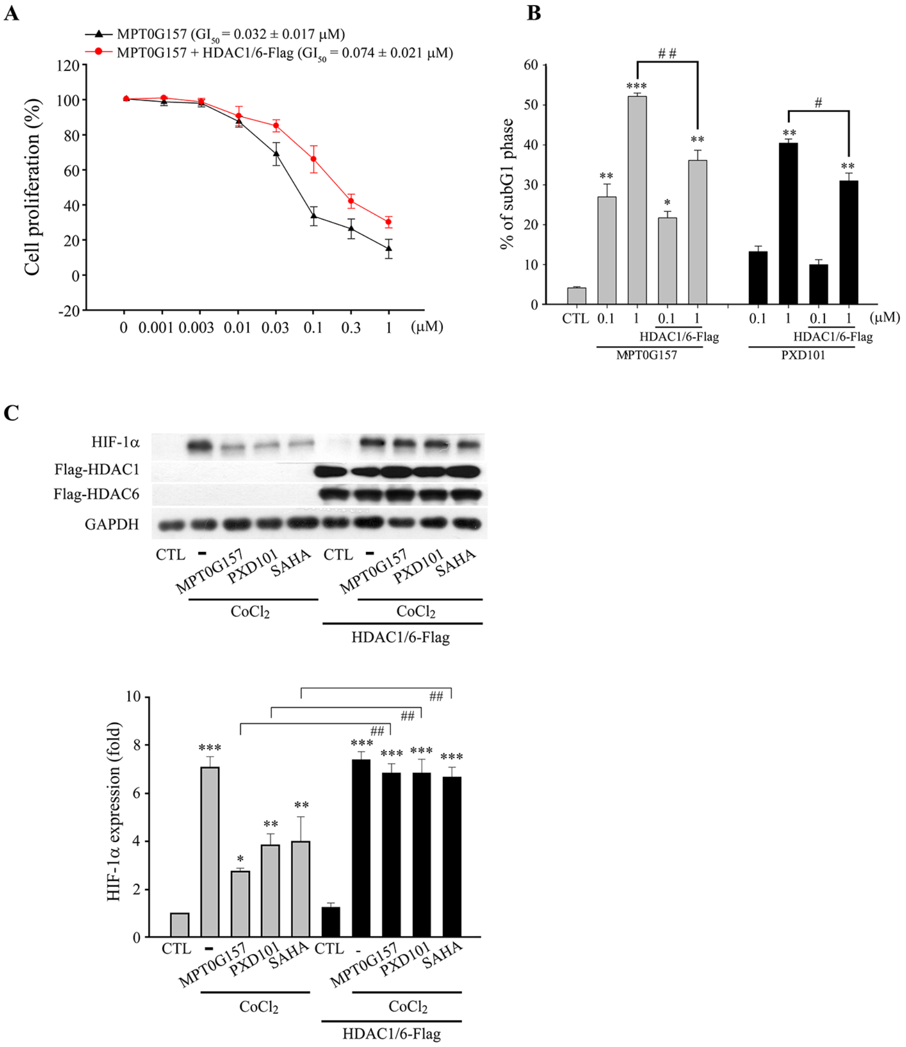

Supplementary Figure 2: Overexpression of HDAC1/HDAC6 reduced MPT0G157-mediated cell proliferation inhibition, apoptosis and HIF-1α suppression. A. HCT116 cells were co-transfect with HDAC1- and HDAC6-flag plasmids 1 μg for 3 h prior to treat indicated concentrations of MPT0G157 for 48 h. Cell proliferation was evaluated by SRB assay. **B.** Cells that were transfected with vectors encoding for both HDAC1 and HDAC6 as in (A) were incubated for 24 h with or without MPT0G157 and PXD101 as indicated concentrations, fixed and then stained with propidium iodide to analyze the DNA contents by flow cytometry. **C.** Cells transfected as in (A) were incubated with MPT0G157, PXD101 or SAHA (0.1 μM) for 20 h prior to Cobalt(II) chloride (300 μM) treatment for another 4 h, and total cell lysates were subjected to western blotting for the HIF-1α, flag-HDAC1, flag-HDAC6 and GAPDH antibodies. Results are shown as mean ± SEM from three independent experiments. *$p < 0.05$, **$p < 0.01$, and ***$p < 0.001$ compared with the relevant control group; #$p < 0.05$, ##$p < 0.01$ compared to indicated group.

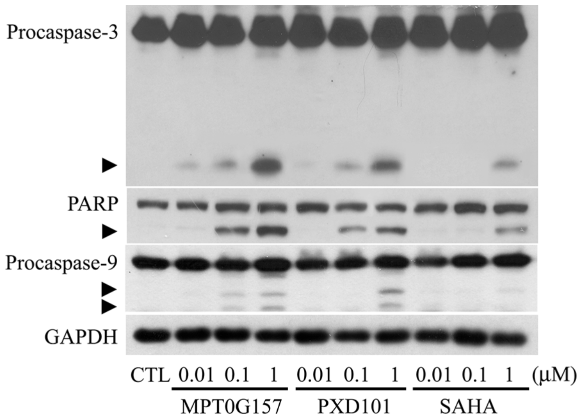

Supplementary Figure 3: MPT0G157 treatment induced a caspase-dependent apoptosis in HCT116 cells. Cells (1×10^6) were incubated for 48 h with or without MPT0G157, PXD101, and SAHA, then total cell lysates were prepared for western blot analysis of the indicated proteins; arrowhead indicated the cleavage form of indicated proteins.

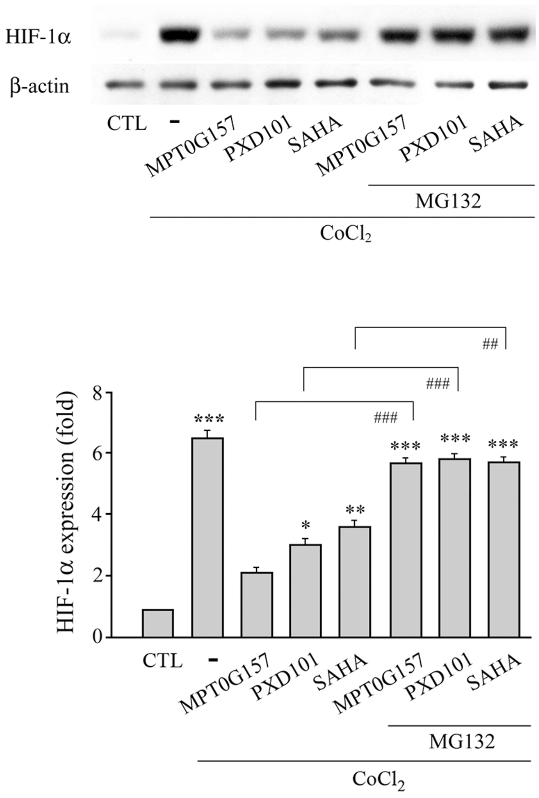

Supplementary Figure 4: Proteasome inhibitor MG132 treatment reversed MPT0G157-mediated HIF-1α degradation. Cells were incubated with MPT0G157, PXD101 or SAHA (0.1 μM) for 16 h and treated with MG132 (10 μM) for 4 h, followed by treatment with Cobalt(II) chloride (300 μM) for another 4 h, total cell lysates were subjected to western blotting for the HIF-1α and β-actin. Results are shown as mean ± SEM from three independent experiments. *$p < 0.05$, **$p < 0.01$, and ***$p < 0.001$ compared with control group; ##$p < 0.01$ and ###$p < 0.001$ compared to indicated group.
